# Supplementary material for: The risk factors for treatment-related mortality within first three months after kidney transplantation
Source: PLoS One. 2020 Dec 10;15(12):e0243586. doi: 10.1371/journal.pone.0243586 (PMC7728215; doi:10.1371/journal.pone.0243586)
Supplement: Attachment — Submitted filename: Response to Reviewers.doc [file pone.0243586.s001.doc]

**Journal requirements:**

**1. Please ensure that your manuscript meets PLOS ONE's style requirements, including those for file naming. The PLOS ONE style templates can be found at**

**https://journals.plos.org/plosone/s/file?id=wjVg/PLOSOne_formatting_sample_main_body.pdf and**

**https://journals.plos.org/plosone/s/file?id=ba62/PLOSOne_formatting_sample_title_authors_affiliations.pdf**

- We have checked the PLOS ONE style templates and confirmed our manuscript to meet requirements. The file naming also checked.

**2. Please include the date(s) on which you accessed the databases or records to obtain the data used in your study.**

🡺 We accessed the Korean Network for Organ Sharing database on June 5, 2020 to obtain additional information for the revision of a previously reported manuscript associated with treatment-related mortality in kidney transplantation. We found a number of important factors for treatment-related mortality, which could not be integrated in the previous study. Therefore, we have persistently accessed the Korean Network for Organ Sharing database extracted from 40 medical centers to identify the risk factors including routine laboratory data until September 23, 2020. We have added the dates and contact information for a data access committee to the revised **Data accessibility statement** section (page 20, lines 349 to 353) as follows.

“We had accessed the Korean Network for Organ Sharing database from June 5, 2020 to September 23, 2020. Contact information for a data access committee is listed as follows: National Organ and Blood Management Institute of the Ministry of Health and Welfare, Tel: 82-2-2628-3602; Official internet site: <https://www.konos.go.kr/konosis>.”

**3. Please describe any inclusion and exclusion criteria used to determine your final cohort.**

🡪 As indicated by the reviewer, we have described inclusion and exclusion criteria, which are similar to our previous study (reference 16) in the revised **Materials and Methods** section (page 5, lines 98 to 101) as follows.

“This study included all patients enrolled for kidney transplantation in the KONOS system of the KCDC between January 2002 and December 2016. We excluded patients who did not have complete demographic information and who concurrently underwent other organ transplantations [16].”

**4. We note that you have stated that you will provide repository information for your data at acceptance. Should your manuscript be accepted for publication, we will hold it until you provide the relevant accession numbers or DOIs necessary to access your data. If you wish to make changes to your Data Availability statement, please describe these changes in your cover letter and we will update your Data Availability statement to reflect the information you provide.**

- We have checked the deposited data in Harvard Dataverse. The accessible DOI provided by the Harvard Dataverse was <https://doi.org/10.7910/DVN/G4OLYV> described in the **Data accessibility statement** section (page 20, lines 348 to 349) as follows. We have published the dataset immediately and the certificate have been attached to the revised cover letter.

“The data involved in this study have been deposited in Harvard Dataverse, and are accessible through https://doi.org/10.7910/DVN/G4OLYV.”

**Response to the reviewer’s comments**

1. **Is the manuscript technically sound, and do the data support the conclusions?**

**Reviewer #1: Yes**

**Reviewer #2: Yes**

- We have corrected and checked that the revised manuscript described a technically sound piece of scientific research and that the data supported the conclusions.

**2. Has the statistical analysis been performed appropriately and rigorously?**

**Reviewer #1: Yes**

**Reviewer #2: Yes**

- We have checked that the statistical analysis has been conducted appropriately and rigorously.

**3. Have the authors made all data underlying the findings in their manuscript fully available?**

**Reviewer #1: Yes**

**Reviewer #2: Yes**

- According to the journal requirements, we have provided the accessible DOI from the Harvard Dataverse for deposited data. Contact information for a data access committee and the dates obtaining the data were described in the revised Data accessibility statement section. We have added these statement to the revised cover letter.

**4. Is the manuscript presented in an intelligible fashion and written in standard English?**

**Reviewer #1: Yes**

**Reviewer #2: Yes**

- We used manuscript editing service before submission.

5. **Review Comments to the Author**

Please use the space provided to explain your answers to the questions above. You may also include additional comments for the author, including concerns about dual publication, research ethics, or publication ethics. (Please upload your review as an attachment if it exceeds 20,000 characters)

**Response to reviewer #1’s comments**

**Treatment-related mortality (TRM) after renal transplantation is a concept different from disease-related mortality and appeared to be a very prevalent entity.**

**Strengths:**

**- There are scarce data in scientific literature about TRM within 1 or 3 months after kidney transplantation.**

**- Authors collected an important amount of data from a very large cohort of patients using a national population based database, which included information about a total of 16,073 kidney recipients.**

**This article would be a valuable contribution to the medical literature to encourage further discussion on this entity.**

**The writing is clear and easily understandable.**

**The Authors have worked hard to improve this article, and they have met all criticisms raised by referees.**

**I feel the manuscript is now suitable for publication in Plos One.**

**Probably a better title could include the concept of an early mortality after renal transplant, and could exclude the origin of the database and the years of observation (“using the Korean Network for Organ Sharing Database, 2002 to 2016”). This might encourage the reader more.**

**For example,**

**The risk factors for early treatment-related mortality in kidney transplantation - A nationwide cohort study**

**Or The risk factors for treatment-related mortality within first three months after kidney transplantation.**

**🡪 Response 1**: As suggested by reviewer, we have changed the title from **“The risk factors for treatment-related mortality in kidney transplantation using the Korean Network for Organ Sharing Database, 2002 to 2016”** to **“The risk factors for treatment-related mortality within first three months after kidney transplantation”** in the revised manuscript.

**Response to reviewer #2’s comments**

**The paper entitle " The risk factors for treatment-related mortality in kidney transplantation using the Korean Network for Organ Sharing Database, 2002 to 2016", is a well written manuscript, the data is well analysed and proper statistical analysis is applied. All the significant outcomes were well discussed and very comprehensive conclusion was made, I highly recommend this paper for publication.**

**🡪 Response 2**: Thank you very much for taking the time to review our manuscript. We are very pleased to receive your valuable comment for our manuscript. We hope it will contribute to better outcome of kidney recipients.

While revising your submission, please upload your figure files to the Preflight Analysis and Conversion Engine (PACE) digital diagnostic tool, https://pacev2.apexcovantage.com/. PACE helps ensure that figures meet PLOS requirements. To use PACE, you must first register as a user. Registration is free. Then, login and navigate to the UPLOAD tab, where you will find detailed instructions on how to use the tool. If you encounter any issues or have any questions when using PACE, please email PLOS at figures@plos.org. Please note that Supporting Information files do not need this step.

- We have uploaded our figure files (Fig 1.tif, and Fig 2.tif) to the PACE digital diagnostic tool to meet PLOS requirements. The preview files (Preview_20201028233814619.pdf, and Preview_20201028233901869.pdf) were generated and checked.
